# Hair repigmentation as a clinical sign of underlying basal cell carcinoma and melanoma

**DOI:** 10.1016/j.jdcr.2026.05.053

**Published:** 2026-05-28

**Authors:** Josefina Gazmuri, Juliana Kida Ikino, Matheus Alves Pacheco, Gabriella Di Giunta Funchal, Alejandra Villarroel, Erica C. Koch Hein, Ashfaq A. Marghoob, Cristian Navarrete-Dechent

**Affiliations:** a Department of Dermatology, Escuela de Medicina, Pontificia Universidad Católica de Chile, Santiago, Chile; b Department of Dermatology, Universidade Federal de Santa Catarina, Florianopolis, SC, Brazil; c Department of Pathology, Escuela de Medicina, Pontificia Universidad Católica de Chile, Santiago, Chile; d Red de Salud UC CHRISTUS, Santiago, Chile; e Melanoma and Skin Cancer Unit, Escuela de Medicina, Pontificia Universidad Católica de Chile, Santiago, Chile; f Department of Hematology and Oncology, School of Medicine, Pontificia Universidad Católica de Chile, Santiago, Chile; g Dermatology Service, Department of Medicine, Memorial Sloan Kettering Cancer Center, New York, New York

**Keywords:** basal cell carcinoma, hair repigmentation, melanoma, skin cancer

## Introduction

Hair greying has long been considered an irreversible process driven by multiple biological mechanisms.^1^ Interestingly, several factors have been associated with hair repigmentation, including medications (eg, monoclonal antibodies, tyrosine kinase inhibitors, immunomodulators), micro-injury (eg, Mohs micrographic surgery), radiotherapy, localized infection (eg, herpes zoster), and underlying cutaneous malignancies.^1^, ^2^, ^3^, ^4^ We describe the first documented case of hair repigmentation associated with basal cell carcinoma. Additionally, we present the first occurrence reported in melanoma superficial spreading subtype, thereby expanding the spectrum of previously published cases.

## Case reports

### Case 1

**Fig 1:**
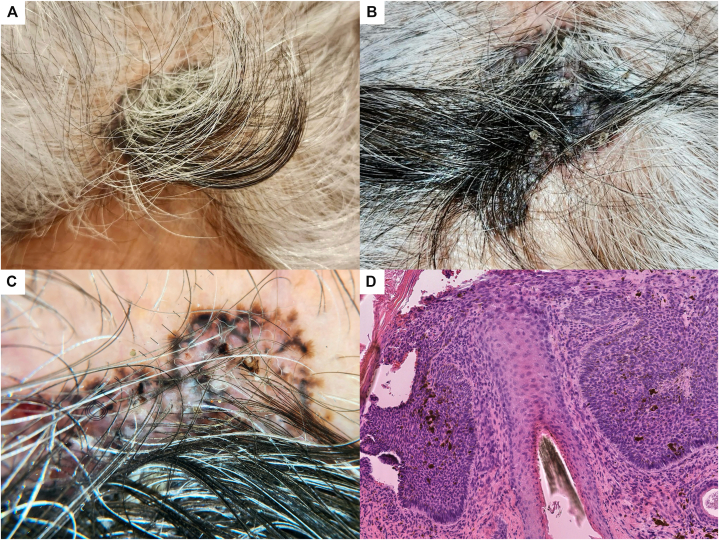
Hair repigmentation overlying a nodular basal cell carcinoma (BCC). **A,** Localized tuft of black hair arising within previously white hair in the left frontal scalp of a 78-year-old woman. **B,** Well-demarcated dark brown 6 × 4 cm plaque underlying repigmented hair. **C,** Dermoscopy showing a central pink background with shiny white blotches and strands, and peripheral dark brown spoke wheel-like structures. **D,** Histopathology revealing a pigmented nodular BCC infiltrating the reticular dermis, with a pigmented hair shaft and no evidence of tumor follicular involvement (H&E, 400×).

A 78-year-old woman developed a tuft of black hair within previously white hair on the left frontal scalp over a pigmented tumor, evolving over approximately 20 months (Fig 1, *A*). Her regular medications were atenolol, olmesartan, and zopiclone. Clinical examination revealed a well-demarcated dark brown 6 × 4 cm plaque underlying the pigmented hair (Fig 1, *B*). Dermoscopy showed a central pink background with shiny white blotches and strands, and peripheral dark brown spoke wheel-like structures (Fig 1, *C*). Histopathological analysis confirmed a pigmented nodular basal cell carcinoma, as well as a pigmented hair shaft devoid of follicular tumor involvement (Fig 1, *D*).

### Case 2

**Fig 2:**
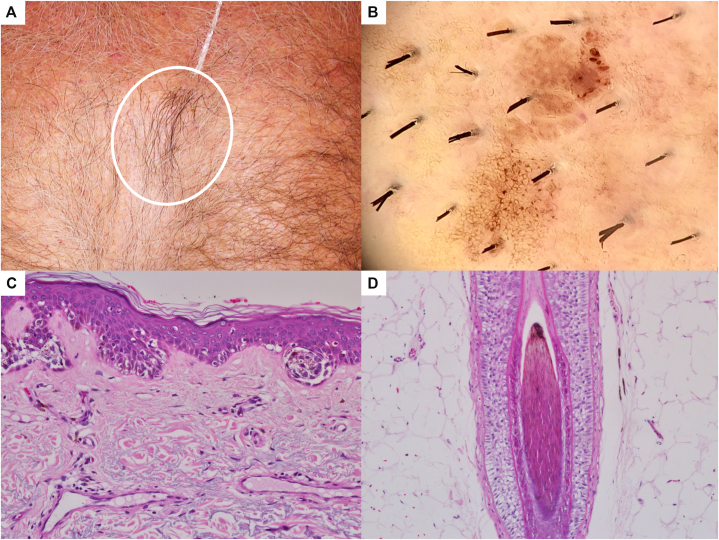
Hair repigmentation overlying a superficially spreading melanoma in situ (MIS). **A,** Localized tuft of repigmented dark hair (*white circle*) on the mid-chest of a 66-year-old man. **B,** Dermoscopy of the underlying 7 × 3 mm brown macule revealed asymmetry in colors and structures, a multicomponent pattern, atypical network in the lower area and atypical peripheral globules in the upper area of the lesion. **C,** Histopathological examination revealed proliferation of atypical melanocytes arranged in irregular nests (H&E, 400×). **D,** Histopathology demonstrating a hair follicle with dendritic melanocytes within the bulb region, with evident pigmentation of the hair shaft and no tumor follicular involvement (H&E, 400×).

A 66-year-old man presented with a 2-year history of spontaneous chest hair repigmentation over a brown lesion. His only medication was rosuvastatin for dyslipidemia. Clinical examination showed a tuft of dark hair in the mid-chest region overlying a 7 × 3 mm irregular brown macule (Fig 2, *A*). Dermoscopy exhibited asymmetry of colors and structures, a multicomponent pattern, and atypical peripheral globules confined to the upper area (Fig 2, *B*). Histopathological examination confirmed a superficial spreading melanoma in situ (Fig 2, *C*). The hair shaft exhibited evident pigmentation and no tumor follicular involvement (Fig 2, *D*).

## Discussion

### Reported cases and epidemiology

• To date, 15 cancer-associated hair repigmentation cases have been reported in the literature.^5^, ^6^, ^7^, ^8^, ^9^, ^10^, ^11^, ^12^, ^13^, ^14^, ^15^, ^16^, ^17^, ^18^, ^19^
• Most cases are associated with melanoma, including lentigo maligna, amelanotic, desmoplastic, and nodular subtypes. One case has been reported in association with microcystic adnexal carcinoma.
• Our cases increase the total number of published cases to 17 (Table I), representing the first reports involving basal cell carcinoma and superficial spreading melanoma.

• Overall, 53% of cases occurred in men.
• Cases predominantly occur on the scalp of elderly individuals (mean age 78 years; range 56-91 years), likely reflecting chronic sun exposure and age-associated hair greying, which provides visual contrast for the repigmented hairs to be clinically apparent.^14^^,^^17^

**Table I:** Overview of reported cases of cancer-associated hair repigmentation

Study, year	Age (y)	Sex	Lesion size (mm)	Anatomical site	Repigmentation pattern	Histopathological diagnosis	Depth (mm)	Extension/spread	Treatment	Outcome/follow-up
Dummer, 2001^5^	76	F	NR	Lateral scalp	Gray → brown/black	MIS, LM type	MIS	Upper hair follicle involvement	Radiotherapy	Hair grew back gray
Inzinger, 2013^6^	91	F	8 + satellite lesions	Occipital scalp	White → red → black → light brown	Melanoma, NOS	3.5	Follicular involvement; lymph node metastasis	1° surgical excision	Local recurrence + lymph node metastasis at 10 mo
									2° surgical excision of local recurrence and lymph node + radiotherapy	No recurrence at 4-mo follow-up
Rahim, 2013^7^	82	F	45 × 55	Parietal scalp	Gray → strawberry blonde	Amelanotic desmoplastic melanoma + surrounding MIS, LM type	7	NR	Wide local excision	No recurrence at 6-y follow-up
Tiger, 2014^8^	88	F	65 × 30	Superior scalp	White → brown	MIS, LM type	MIS	Dermoepidermal junction; follicular involvement	NR	NR
Oza, 2015^9^	58	F	19 × 9 + satellite lesions	Vertex scalp	Grey → brown/blonde	Nodular melanoma	4.4	Dermal-subcutaneous junction; lymph node metastasis; pulmonary metastasis	Wide local excision + radiotherapy + chemotherapy (temozolomide)	Close follow-up with repeat PET/CT monitoring
Amann, 2016^10^	91	F	40 × 30	Occipital scalp	Grey → black	MIS, LM type	MIS	Follicular epithelium involvement	Staged excision	NR
Chan, 2019^11^	80	M	30 × 25	Vertex scalp	Grey → brown/black	MIS, LM type	MIS	Follicular involvement	Wide local excision	No recurrence after 1-y follow-up
Chew, 2019^12^	75	M	30 × 30	Parietal scalp	White/grey → brown/black	MIS and invasive MM, LM type	1	Superficial hair follicle involvement; eccrine duct extension	Wide local excision	NR
Lackey, 2019^13^	86	M	150 × 80	Vertex and occipital scalp	White → black	MIS, LM type	MIS	NR	Topical imiquimod 5% cream	Complete response; no recurrence at 17-mo follow-up
López-Sánchez, 2020^14^	56	M	NR	Vertex scalp	White → brown/black	MIS, LM type	MIS	Follicular involvement	Surgical excision	No recurrence at 12-mo follow-up
Hasegawa, 2021^15^	80	M	13.8 × 5.8	Parietal scalp	Grey → brown/black	Amelanotic melanoma	NR	Perifollicular infiltration; in-transit metastasis; cervical lymph nodes metastasis	Pembrolizumab 2 mg/kg every 3 wk for 5 cycles	Hair returned grey, reduced size of primary tumor; disappearance of in-transit metastasis and cervical lymph node metastasis; no recurrence at 12-mo follow-up
Gessler, 2022^16^	85	M	5	Parietal scalp	White → brown	MIS, LM type	MIS	Follicular involvement	Staged excision	Clear margins
Ly, 2023^17^	85	M	50	Parietal scalp	White → black	Melanoma, NOS	1	Deep follicular involvement	Staged excision	No recurrence at 12-mo follow-up
Saal, 2023^18^	74	M	NR	Midline scalp	White/grey → brown	Microcystic adnexal carcinoma	NR	Dermal and subcutaneous extension; perineural invasion	Slow Mohs	NR
Doan, 2023^19^	82	F	40 × 30	Vertex scalp	Grey → reddish-brown	MIS, NOS	MIS	Follicular infundibulum involvement	Wide local excision	Clear margins
Current case 1, 2025	78	F	60 × 40	Frontal scalp	White → black	Nodular BCC	≥1	No follicular involvement	Lost to follow-up	Lost to follow-up
Current case 2, 2025	66	M	7 x 3	Chest	Grey → brown/black	MIS, superficial spreading type	MIS	No follicular involvement	Wide local excision	No recurrence at 24-mo follow-up

*BCC*, Basal cell carcinoma; *LM*, lentigo maligna; *MIS*, melanoma in situ; *NOS*, not otherwise specified; *NR*, not reported; *PET/CT*, positron emission tomography/computed tomography.

### Hair greying pathophysiology

**Fig 3:**
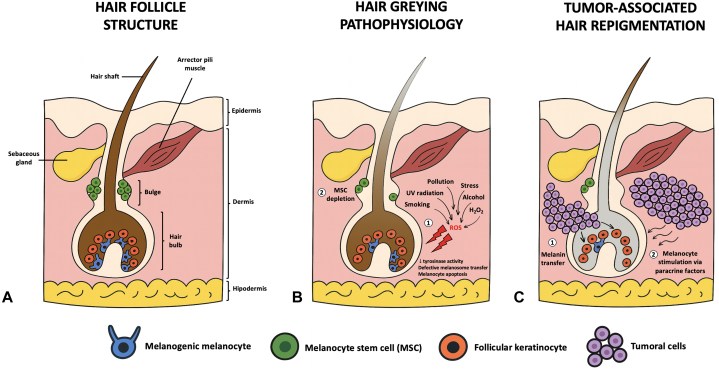
Hair follicle structure, hair greying pathophysiology, and proposed mechanisms of tumor-associated hair repigmentation. **A,** The hair follicle contains a specialized pigmentary unit in which melanogenic melanocytes synthesize melanin and transfer it to adjacent follicular keratinocytes, thereby pigmenting the hair shaft. During catagen, differentiated melanogenic melanocytes enter apoptosis. In the following anagen, melanocyte stem cells (MSCs) in the bulge differentiate, migrate, and repopulate the hair bulb. **B,** With ageing, hair greying begins as melanogenesis gradually declines due to reduced tyrosinase activity, defective melanosome transfer, melanocyte apoptosis, and cumulative oxidative stress from reactive oxygen species. Subsequently, MSCs become depleted, eliminating the reservoir required to restore melanogenic melanocytes. **C,** Two models may explain tumor-associated repigmentation: (1) tumor cells infiltrate the hair follicle and directly transfer melanin to follicular keratinocytes, and (2) paracrine signals from tumor cells reactivate melanogenesis within melanogenic melanocytes without follicular invasion.

• Hair greying is a complex, multifactorial process regulated by diverse intrinsic and extrinsic factors (Fig 3, *A* and *B*).^3^

• It begins with a gradual decline in melanogenesis within melanogenic melanocytes in the hair follicle pigmentary unit, driven by reduced tyrosinase activity, defective melanosome transfer, melanocyte apoptosis, and oxidative stress.^3^^,^^20^
• Oxidative stress derives from the accumulation of reactive oxygen species, produced by hydrogen peroxide build-up, ultraviolet radiation, environmental pollution, emotional stress, alcohol consumption, and smoking.^3^
• Melanocyte stem cells serve as a reservoir for melanogenic melanocytes, differentiating to restore the hair follicle pigmentary unit at the start of each new anagen phase.^1^
• Following the initial decline in melanogenesis, melanocyte stem cells become depleted, resulting in an impaired melanocyte replenishment.^20^
• Melanocyte stem cell depletion is considered the major determinant of the potential irreversibility of hair greying, with additional contributions from genetic, metabolic, and endocrine factors.^1^^,^^20^

### Proposed mechanisms of cancer-associated hair repigmentation

• The exact mechanisms underlying cancer-associated hair repigmentation remain unclear.
• Two main hypothesis have been proposed (Fig 3, *C*):
  1. Infiltrating cancer cells directly transfer melanin to follicular keratinocytes, supported by cases demonstrating tumor involvement of the hair bulb on histopathology.^17^
  2. Cancer cells activate resident bulb melanocytes through paracrine melanocyte-stimulating factors, possibly accounting for cases lacking histopathological signs of hair bulb infiltration.^17^
• In both our cases, the superficial nature of the tumors and absence of hair unit tumor invasion on histopathology support a paracrine mechanism as the most likely explanation.

### Clinical implications

• The presence of a solitary tuft of repigmented hair in a hair-bearing area should prompt careful examination of the underlying skin.
• Lesions on the scalp may be overlooked due to limited visibility, and are often identified by dermatologists, relatives, or hairdressers rather than patients themselves.
• Similarly, poliosis circumscripta (a localized patch of white hair among pigmented hair) may represent a clinical marker of underlying melanoma and should also raise concern for malignancy.^21^
• Tumor cells might induce changes in the local microenvironment that trigger hair repigmentation.
• Further investigation of underlying cancer-associated hair repigmentation mechanisms could provide insights into treatments for hair greying, with potential benefits for patient quality of life.

## Conflicts of interest

None disclosed.
